# Supplementary figures and images for: Fraction of plasma exomeres and low-density lipoprotein cholesterol as a predictor of fatal outcome of COVID-19
Source: PLoS One. 2023 Feb 9;18(2):e0278083. doi: 10.1371/journal.pone.0278083 (PMC9910704; doi:10.1371/journal.pone.0278083)

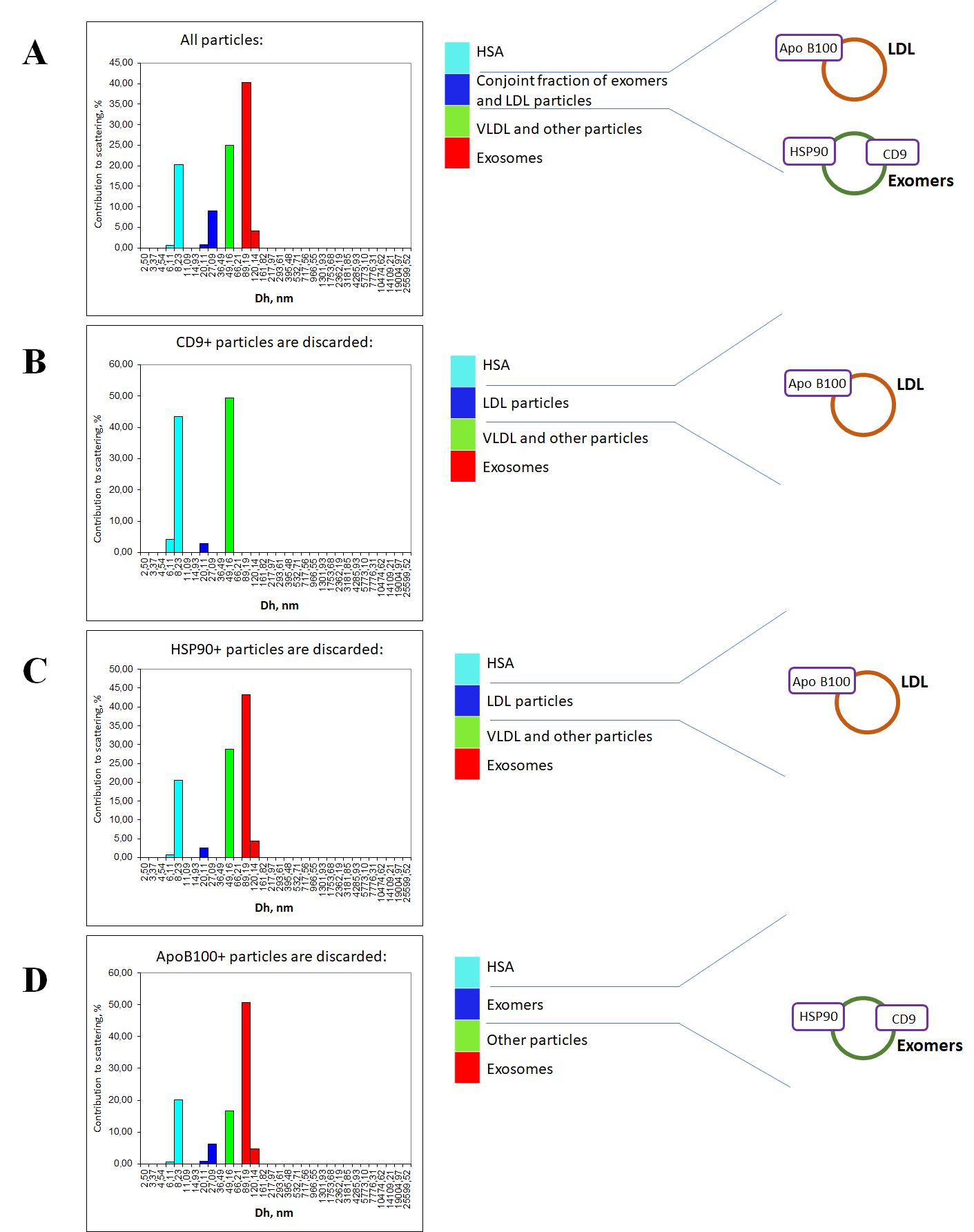

Supplement: S1 Fig — Principle of particle count estimation by dynamic light scattering (DLS) combined with immunoadsorption: A. Analysis of unprocessed plasma, B. Analysis of plasma after CD9 positive particles (exosomes, exomeres) depletion, C. Analysis of plasma after HSP90 positive particles (exomeres) depletion, D. Analysis of plasma after apolipoprotein B100 positive particles (LDLs, VLDLs) depletion. Abbreviations on the figure: ApoB100—apolipoprotein B100, Dh–hydrodynamic diameter, HSA–human serum albumin, LDL–low density lipoproteins, VLDL–very low-density lipoproteins. (TIF) [file pone.0278083.s001.tif]

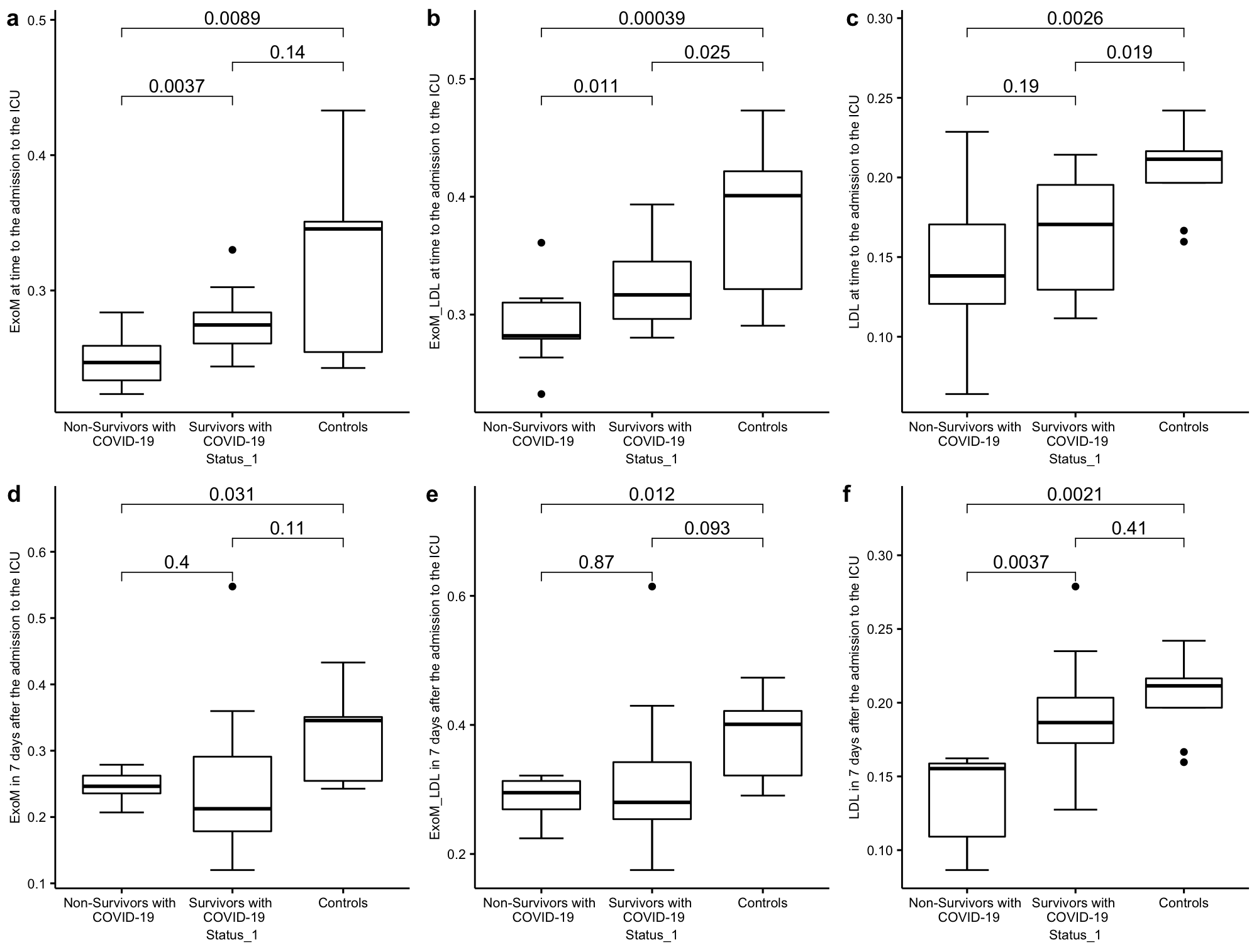

Supplement: S2 Fig — Relative level of plasma exomeres with concentration of TC bound by LDL for patients with COVID-19 infected by Alpha variant: A. ExoM (admission to the ICU), B. ExoM_LDL (admission to the ICU), C. LDL (admission to the ICU), D. ExoM (7 days after admission to the ICU), E. ExoM_LDL (7 days after admission to the ICU), F. LDL (7 days after admission to the ICU). (TIF) [file pone.0278083.s002.tif]

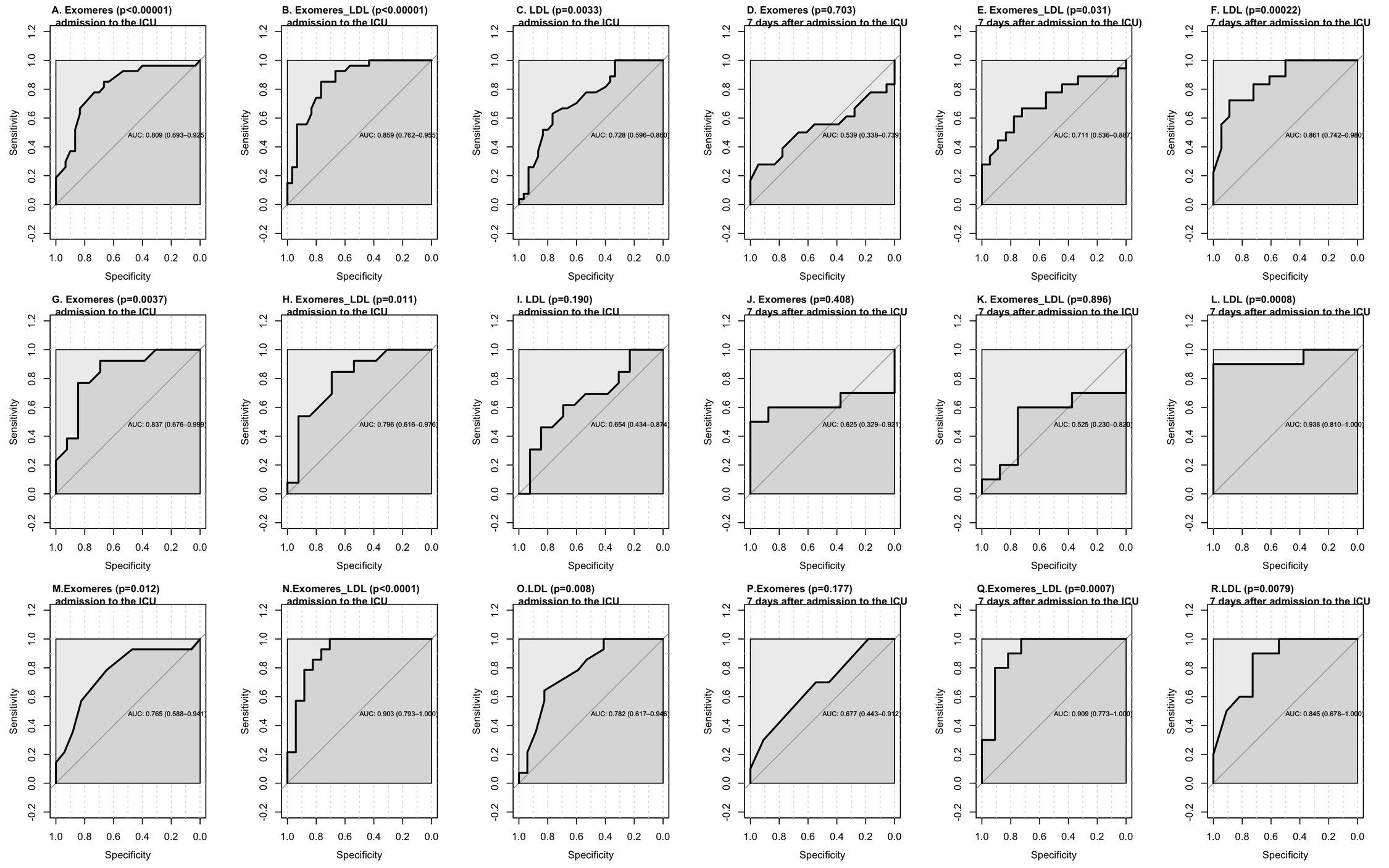

Supplement: S3 Fig — ROC analysis for in blood plasma for all patients with COVID-19 (A-F): A. ExoM (admission to the ICU), B. ExoM_LDL (admission to the ICU), C. LDL (admission to the ICU), D. ExoM (7 days after admission to the ICU), E. ExoM_LDL (7 days after admission to the ICU), F. LDL (7 days after admission to the ICU); for patients with COVID-19 infected by the Alpha variant (G-L): G. ExoM (admission to the ICU), H. ExoM_LDL (admission to the ICU), I. LDL (admission to the ICU), J. ExoM (7 days after admission to the ICU), K. ExoM_LDL (7 days after admission to the ICU), L. LDL (7 days after admission to the ICU); for patients with COVID-19 infected by the Delta variant (M-Q): M. ExoM (admission to the ICU), N. LDL (admission to the ICU), O. LDL (admission to the ICU), P. ExoM (7 days after admission to the ICU), Q. ExoM_LDL (7 days after admission to the ICU), R. LDL (7 days after admission to the ICU). (TIF) [file pone.0278083.s003.tif]
