# Supplementary material for: Fraction of plasma exomeres and low-density lipoprotein cholesterol as a predictor of fatal outcome of COVID-19
Source: PLoS One. 2023 Feb 9;18(2):e0278083. doi: 10.1371/journal.pone.0278083 (PMC9910704; doi:10.1371/journal.pone.0278083)
Supplement: S1 File — (PDF) [file pone.0278083.s004.pdf]

Plasma cytokine profile in the studied patients with COVID-19 with different outcome (survivor, non-survivor) infected by delta variant of SARS-CoV-2 at the time to admission to the ICU and in the control group

Plasma cytokine profile was estimated in subgroups of patients with COVID-19 - survivors and non-survivors - at the time of admission to the ICU, as well as the control group. The concentrations of plasma cytokines in the studied groups are presented in Table 1. We found an increased concentration of pro-inflammatory cytokines IL-15, IL-27 in non-survivors when compared with controls and survivors (IL-15: p=0.00014, p=0.00096, respectively; IL-27: p<0.0001, p=0.011, respectively). Secretion of IL-10 and CCL20/MIP3α was increased in non-survivors compared to controls (p=0.0027, p=0.012, respectively). Also, an increase in the concentration of pro-inflammatory cytokines such as IL-15, IL-6, IL-27 was also shown in the subgroup of survived patients with COVID-19 compared with the control group (p=0.049, p=0.026, p=0.00032, respectively). There were no statistically significant differences between the studied groups when comparing the concentration of other analytes in blood plasma (p>0.05).

Table 1. Cytokine profile of blood plasma of the studied groups at the time of admission to the ICU

Analytes	The concentration of analytes in blood plasma, median (min-max), pg/ml		
	Baseline (Admission to the ICU)		Control group
	Non-survivors	Survivors	
IL-17F	0.039(0.00558-0.164)	0.036(0.0121-0.066)	0.035(0.0072- 0.058)
GM-CSF	0.107(0.018-0.475)	0.11(0.042-0.475)	0.157(0.0039 0.279)
IFNγ	22.06(3.53-85.69)	28.32(9.32-106.689)	26.55(7.12- 41.81)
IL-10	23.079(7.41-197.08)<br>**p=0.0027***<br>(*p=0.0081*[a])	15.23(8.54-43.84)	12.61(4.435-17.55)
CCL20/MIP3α	55.72(24.56- 1723.642)<br>**p=0.012***<br>(*p=0.036*[a])	37.04(24.56-87.47)	35.35(6.02 53.21)
IL-12(p70)	9.68(2.04-32.459)	10.22(2.047- 24.83)	13.22(4.710-20.942)
IL-13	58.87(8.080-106.856)	42.64(7.324- 98.20)	54.18(10.965-77.750)
IL-15	27.19(7.25- 51.87)<br>**p=0.00014***<br>(*p=0.00042*[a])<br>**p=0.00096****<br>(*p=0.00288*[a])	18.92(11.54-33.90)<br>**p=0.049***<br>(*p=0.147*[a])	10.94(2.720916 17.178)
IL-17A	12.20(3.582-63.86)	12.60(0.492-28.12)	12.78(0.492519.57)

IL-22	0.648(0.036-1.479)	0.648(0.344-1.082)	0.61(0.255-0.862)
IL-9	51.14(12.088-124.746)	49.24(12.08-125.715)	67.56(18.71- 97.768)
IL-1β	7.06(1.43-23.983)	5.63(0.536-14.70)	6.70(0.536-10.805)
IL-33	36.78(1.680-89.866)	36.78(13.45-77.43)	43.18(13.457-58.65)
IL-2	13.74(0.0297-105.212)	13.74(6.596-37.128)	15.80(0.029- 27.192)
IL-21	31.59(6.149-95.788)	28.19(8.20-63.75)	35.71(10.189-49.578)
IL-4	0.346(0.024-1.4260)	0.363(0.0244-1.1017)	0.379(0.0244 0.587)
IL-23	3.15(0.359-8.69)	3.64(1.144-7.589)	3.69(1.286-5.451)
IL-5	15.10(0.142-77.59)	20.17(6.17- 57.79)	21.96(7.2337.7932)
IL-6	35.47(6.579-100)	35.71(1.7 127.677) **p=0.026*** (*p=0.078* [a])	16.80(1.706- 34.20)
IL-17E/IL-25	0.122(0.0035- 0.212)	0.111(0.0035-0.177)	0.097(0.04- 0.122)
IL-27	1.98(0.900-2.7) **p<0.0001*** (*p<0.0001* [a]) **p=0.011**** (*p=0.033*[a])	1.38(1.011-2.57) **p=0.00032*** (*p=0.00096* [a])	0.928(0.688-1.21)
IL-31	0.0477(0.010-0.202)	0.0606(0.007-0.141)	0.061(0.013-0.0971)
TNFα	31.32(16.60-99.21)	33.12(16.607-63.29)	22.49(12.20-31.58)
TNFβ	0.08(0.0164-0.166)	0.059(0.0218-0.182)	0.072(0.003-0.1365)
IL-28A	0.166(0.048-0.419)	0.178(0.0077-0.644)	0.194(0.0321-0.317)

p* - compared with the control group

p**- compared with survived patients with COVID-19

p[a] – p-value with Bonferroni correction for multiple comparisons
