# Supplementary material for: Fraction of plasma exomeres and low-density lipoprotein cholesterol as a predictor of fatal outcome of COVID-19
Source: PLoS One. 2023 Feb 9;18(2):e0278083. doi: 10.1371/journal.pone.0278083 (PMC9910704; doi:10.1371/journal.pone.0278083)
Supplement: S1 Table — (DOCX) [file pone.0278083.s005.docx]

**S1 Table.** **Primers and probe sequences for estimation expression levels of the studied genes**

| **Gene** | Sequence 5’-3’ | | |
| --- | --- | --- | --- |
|  | Forward | Reverse | Probe |
| ***LDLR*** | 3’-TGGAAAAGTGGCCAGTGGAT-5’ | 3’-ATGCAGCGGTTGACACGG-5’ | 3’(FAM)- CATGAATTATGTCTGTCACCTGC-(BHQ1)-5’ |
| ***PPARG*** | 5’-GATGTCTCATAATGCCATCACGTT-3’ | 5’-GGATTCAGCTGGTCGATATCACT-3’ | 5’(FAM)-CCAACAGCTTCTCCTTCTCGGCCTG - 3’(RTQ1) |
| ***LRP6*** | 5’- TGATGTGACAGACAACCGAAT-3’ | 5’- GCCATGCCTTCTGGATAATC-3’ | 5’-FAM- CACTCAAGACCATCAGCAGAGC-BHQ1-3’ |
| ***CD36*** | 5’- TCAGTTGGAACAGAGGCTGA-3’ | 5’ - TGAATTGAGGATCATTTGAACA -3’ | 5’(FAM)- CTCAATCTGGCTGTGGCAGCTG -(BHQ1)-3’ |
| ***STAB1*** | 5’- GCCGCCTGATCTACCTCTTC-3’ | 5’-TGGCCGTGGTTGTAGATGT-3’ | 5’(FAM)- CAGCGGGTCTCTCTAAACTGCAG-(BHQ1)-3’ |
| ***ANXA2*** | 5’- GATATTGCCTTCGCCTACCA -3’ | 5’- TCAATAGGCCCAAAATCACC -3’ | 5’(FAM)- CAAAAAGGAACTTGCATCAGCACTGA-(BHQ1)- |
| ***RPLPO*** | 5’-GACTTCACATGGGGCAATGG-3’ | 5’-GATCAGGGACATGTTGCTGG-3’ | 5’-(ROX)-CAATAAGGTGCCAGCTGCTGC(BHQ2)-3’ |
| ***ACTB*** | 5’-CGTGCTGCTGACCGAGG-3’ | 5’-ACAGCCTGGATAGCAACGTACA-3’ | 5’-(HEX) CCAACCGCGAGAAGATGACCCAGAT(BHQ1)-3’ |
