# Supplementary material for: Fraction of plasma exomeres and low-density lipoprotein cholesterol as a predictor of fatal outcome of COVID-19
Source: PLoS One. 2023 Feb 9;18(2):e0278083. doi: 10.1371/journal.pone.0278083 (PMC9910704; doi:10.1371/journal.pone.0278083)
Supplement: S2 Table — (DOCX) [file pone.0278083.s006.docx]

**S2 Table.** **Relative level of plasma exomeres for patients with COVID-19 with concentration of TC bound by LDL**

| Parameters | Lipid spectrum of blood plasma, median (min-max), mmol/l | | | | | | | | | | | | |
| --- | --- | --- | --- | --- | --- | --- | --- | --- | --- | --- | --- | --- | --- |
|  | All patients with COVID-19 | | | | Patients with COVID-19 infected by alpha variant | | | | Patients with COVID-19 infected by delta variant | | | | Control group (N=40) |
|  | Admission to the ICU | | 7 days after admission to the ICU | | Admission to the ICU | | 7 days after admission to the ICU | | Admission to the ICU | | 7 days after admission to the ICU | |  |
|  | Non-survivors (N=34) | Survivors (N=33) | Non-survivors (N=34) | Survivors (N=33) | Non-survivors (N=21) | Survivors (N=18) | Non-survivors (N=21) | Survivors (N=18) | Non-survivors (N=13) | Survivors (N=15) | Non-survivors (N=13) | Survivors (N=15) |  |
| ExoM | 0.247(0.223-0.284)  **p=0.018****  **p=6.3e-05*** | 0.275(0.244-0.330)  **p=0.03**** | 0.246(0.207-0.279)  **p=0.03**** | 0.212(0.120-0.548)  p=0.018* | 0.247(0.223-0.284)  **p=0.0037***  **p=0.0089**** | 0.275(0.244-0.330) | 0.246(0.207-0.279)  **p=0.031**** | 0.212(0.120-0.548) | 0.240 (0.20-0.45)  **p=0.012***  **p=0.034**** | 0.265 (0.20-0.45) | 0.235 (0.19-0.28)  **p=0.037**** | 0.260 (0.22-0.41) | 0.345(0.243-0.433) |
| LDL | 0.135(0.064-0.229)  **p=4.5E-05****  **p=0.0033*** | 0.180(0.112-0.230) | 0.140(0.060-0.200)  **p=8.8e-05****  **p=0.00022***** | 0.191(0.127-0.279)  p=0.062* | 0.138(0.064-0.229)  **p=0.0026**** | 0.170(0.112-0.214)  **p=0.019**** | 0.155(0.086-0.162)  **p=0.0021****  **p=0.0037***** | 0.187(0.127-0.279) | 0.140 (0.08-0.21)  **p=0.008***  **p=0.00021**** | 0.175 (0.11-0.23) | 0.115 (0.06-0.20)  **p=0.0014****  **p=0.0079***** | 0.190 (0.13-0.21) | 0.170(0.090-0.242) |
| ExoM_LDL | 0.270(0.204-0.361)  **p=1.5e-05****  **p=3.6e-06*** | 0.317(0.259-0.473) | 0.281(0.224-0.330)  **p=0.00099****  **p=0.031***** | 0.311(0.175-0.614) | 0.282(0.232-0.361)  **p=0.011***  **p=0.00039**** | 0.317(0.280-0.393)  **p=0.025**** | 0.295(0.224-0.321)  **p=0.012**** | 0.280(0.175-0.614) | 0.248(0.204-0.338)  **P=4.2e-05***  **p=2.8e-05**** | 0.314(0.253-0.473) | 0.271(0.215-0.330)  **p=0.0015****  **p=0.00079***** | 0.318(0.287-0.439) | 0.297(0.147-0.500) |
| ExoS | 0.670(0.641-0.690)  **p=0.003****  **p=0.024*** | 0.661(0.655-0.608)  **p=9e-05**** | 0.670(0.655-0.692)  **p=0.014**** | 0.665(0.633-0.674)  **p=0.00036**** | 0.670(0.641-0.675) | 0.662(0.655-0.670) | 0.665(0.655-0.692) | 0.665(0.633-0.669)  **p=0.082**** | 0.660 (0.66-0.68)  **p=0.081***  **p=0.0018**** | 0.670 (0.66-0.69)  **p=0.00016**** | 0.670 (0.65-0.67)  **p=0.0064**** | 0.670 (0.66-0.68)  **p=0.0001**** | 0.680(0.443-0.712) |

* - compared to survivor with COVID-19 (admission to the ICU)

** - compared to controls

*** - compared to survivor with COVID-19 (7 days after admission to the ICU)
