# Supplementary material for: Fraction of plasma exomeres and low-density lipoprotein cholesterol as a predictor of fatal outcome of COVID-19
Source: PLoS One. 2023 Feb 9;18(2):e0278083. doi: 10.1371/journal.pone.0278083 (PMC9910704; doi:10.1371/journal.pone.0278083)
Supplement: S3 Table — (DOCX) [file pone.0278083.s007.docx]

**S3 Table.** **Correlation analysis between lipid profile parameters and SIC exomeres in patients with COVID-19 at the time of admission to ICU and in control group**

| Parameters | TC | HDL | LDL | TG | TC | HDL | LDL | TG | TC | HDL | LDL | TG |
| --- | --- | --- | --- | --- | --- | --- | --- | --- | --- | --- | --- | --- |
|  | All patients with COVID-19 and control group | | | | Patients with COVID-19 infected by alpha variant | | | | Patients with COVID-19 infected by delta variant | | | |
| ExoM | **r=0.459***  **p=0.011***  **r=0.623****  **p=0.0007****  **r=0.740*****  **p=4.484e-08***** | **r=0.518***  **p=0.003***  **r=0.738****  **p=1.658e-05****  **r=0.310*****  **p=0.052***** | **r=0.391***  **p=0.036***  **r=0.535****  **p=0.005****  **r=0.762*****  **p=1.786e-08***** | r=-0.021*  p=0.913*  r=-0.042**  p=0.840**  r=0.087***  p=0.593*** | r=0.400*  p=0.175*  **r=0.764****  **p=0.004**** | r=0.404*  p=0.171*  **r=0.632****  **p=0.024**** | r=0.287*  p=0.366*  **r=0.065****  **p=0.019**** | r=0.157*  p=0.609*  r=0.033**  p=0.0921** | r=0.232*  p=0.311*  **r=0.486****  **p=0.047**** | **r=0.448***  **p=0.041***  **r=0.715****  **p=0.001**** | r=0.093*  p=0.687*  r=0.321**  p=0.209** | r=0.137*  p=0.555*  r=0.162**  p=0.535** |
| LDL, rlpU | **r=0.781***  **p=3.493e-07***  **r=0.632****  **p=0.0005****  **r=0.900*****  **p=2.944e-15***** | **r=0.600***  **p=0.0005***  r=0.166**  p=0.418**  **r=0.458*****  **p=0.003***** | **r=0.825***  **p=3.803e-08***  **r=0.624****  **p=0.0007****  **r=0.910*****  **p=1.087e-15***** | r=-0.245*  p=0.191*  r=0.291**  p=0.150**  r=0.091***  p=0.577*** | **r=0.703***  **p=0.0073***  **r=0.895****  **p<0.00001**** | **r=0.544***  **p=0.054***  r=0.318**  p=0.289** | **r=0.748***  **p=0.007***  **r=0.895****  **p<0.00001**** | r=-0.123*  p=0.687*  r=0.071**  p=0.821** | **r=0.844***  **p=1.504e-06***  **r=0.387****  **p=0.0125**** | **r=0.593***  **p=4.64e-03***  r=-0.085**  p=0.745** | **r=0.854***  **p=8.258e-07***  **r=0.503****  **p=0.039**** | r=-0.155*  p=0.502*  r=0.211**  p=0.416** |
| ExoM_LDL | **r=0.499***  **p=0.005***  **r=0.847****  **p=4.874e-08****  **r=0.850*****  **p=4.010e-12***** | **r=0.365***  **p=0.047***  **r=0.603****  **p=0.001****  **r=0.386*****  **p=0.014***** | **r=0.486***  **p=0.008***  **r=0.774****  **p=3.484e-06****  **r=0.878*****  **p=2.053e-13***** | r=0.036*  p=0.848*  r=0.184**  p=0.367**  r=0.085***  p=0.601*** | **r=0.761***  **p=0.0025***  **r=0.967****  **p<0.00001**** | r=0.319*  p=0.288*  r=0.505**  p=0.081** | **r=0.741***  **p=0.008***  **r=0.912****  **p<0.00001**** | r=0.222*  p=0.464*  r=0.011**  p=0.978** | r=0.233*  p=0.272*  **r=0.667****  **p=0.0044**** | r=0.370*  p=0.097*  **r=0.573****  **p=0.016**** | r=0.201*  p=0.379*  **r=0.561****  **p=0.0209**** | r=0.107*  p=0.648*  r=0.323**  p=0.207** |
| ExoS | r=-0.171*  p=0.366*  r=0.040**  p=0.845**  r=0.234***  p=0.147*** | r=-0.316*  p=0.089*  r=0.196**  p=0.338**  r=-0.039***  p=0.812*** | r=-0.153*  p=0.428*  r=-0.012**  p=0.954**  r=0.232***  p=0.156*** | r=0.019*  p=0.922*  r=-0.094**  p=0.649**  r=0.037***  p=0.821*** | r=-0.254*  p=0.403*  r=-0.088**  p=0.779** | **r=-0.602***  **p=0.029***  r=0.060**  p=0.849** | r=-0.126*  p=0.699*  r=-0.115**  p=0.709** | r=-0.247*  p=0.415*  r=-0.176**  p=0.566** | r=0.028*  p=0.902*  r=0.207**  p=0.424** | r=0.179*  p=0.437*  r=0.367**  p=0.147** | r=-0.055*  p=0.815*  r=0.166**  p=0.523** | r=0.098*  p=0.673*  r=0.032**  p=0.902** |

* - in non-survivors, ** - in survivors, *** - in controls
