# Supplementary material for: Fraction of plasma exomeres and low-density lipoprotein cholesterol as a predictor of fatal outcome of COVID-19
Source: PLoS One. 2023 Feb 9;18(2):e0278083. doi: 10.1371/journal.pone.0278083 (PMC9910704; doi:10.1371/journal.pone.0278083)
Supplement: S4 Table — (DOCX) [file pone.0278083.s008.docx]

**S4 Table****. Correlation analysis between lipid profile parameters and SIC exomeres in patients with COVID-19 7 days after admission to the ICU**

| Parameters | TC | HDL | LDL | TG | TC | HDL | LDL | TG | TC | HDL | LDL | TG |
| --- | --- | --- | --- | --- | --- | --- | --- | --- | --- | --- | --- | --- |
|  | All patients with COVID-19 and control group | | | | Patients with COVID-19 infected by alpha variant | | | | Patients with COVID-19 infected by delta variant | | | |
| ExoM | r=0.447*  p=0.063*  **r=0.652****  **p=0.002**** | r=0.155*  p=0.540*  **r=0.541****  **p=0.017**** | r=0.353*  p=0.150*  r=0.160**  p=0.526** | r=0.435*  p=0.071*  **r=0.660****  **p=0.002**** | **r=0.883***  **p=0.008***  **r=0.976****  **p=0.00039**** | r=-0.234*  p=0.613*  r=0.622**  p=0.099** | **r=0.857***  **p=0.024***  r=0.678**  p=0.109** | r=0.750*  p=0.066*  **r=0.742****  **p=0.035**** | r=0.146*  p=0.667*  r=0.151**  p=0.698** | r=0.043*  p=0.898*  r=0.303**  p=0.429** | r=-0.043*  p=0.899*  r=-0.529**  p=0.143** | r=0.425*  p=0.193*  r=0.235**  p=0.542** |
| LDL, rlpU | **r=0.763***  **p=0.0002***  r=0.171**  p=0.496** | **r=0.537***  **p=0.022***  r=0.317**  p=0.186** | **r=0.732***  **p=0.0005***  **r=0.916****  **p=9.762e-08**** | r=0.172*  p=0.496*  r=0.351**  p=0.141** | r=-0.054*  p=0.908*  **r=0.695****  **p=0.056**** | r=-0.054*  p=0.908*  **r=0.694****  **p=0.056**** | r=0.393*  p=0.395*  **r=0.821****  **p=0.034**** | **r=0.821***  **p=0.034***  r=0.347**  p=0.399** | **r=0.748***  **p=0.008***  r=0.312**  p=0.413** | **r=0.631***  **p=0.037***  r=0.143**  p=0.713** | **r=0.712***  **p=0.014***  **r=0.878****  **p=0.002**** | r=-0.037*  p=0.915*  r=0.194**  p=0.617** |
| ExoM_LDL | **r=0.827***  **p=2.321e-05***  **r=0.789****  **p=8.296e-05**** | r=0.385*  p=0.114*  **r=0.661****  **p=0.002**** | **r=0.771***  **p=0.0003***  r=0.331**  p=0.179** | r=0.342*  p=0.165*  **r=0.749****  **p=0.0002**** | **r=0.883***  **p=0.00845***  **r=0.976****  **p=0.00039**** | r=-0.414*  p=0.355*  r=0.623**  p=0.099** | **r=0.928***  **p=0.0067***  r=0.678**  p=0.169** | r=0.678*  p=0.109*  **r=0.742***  **p=0.034**** | r=0.665*  p=0.025*  r=0.700**  p=0.044** | r=0.551*  p=0.078*  r=0.700**  p=0.043** | r=0.487*  p=0.128*  r=0.101**  p=0.811** | r=0.227*  p=0.503*  r=0.683**  p=0.051** |
| ExoS | r=0.025*  p=0.921*  r=-0.083**  p=0.735** | r=-0.056*  p=0.528*  r=0.012**  p=0.963** | r=-0.046*  p=0.856*  r=-0.129**  p=0.611** | r=-0.073*  p=0.773*  r=0.012**  p=0.963** | r=-0.180*  p=0.699*  r=-0.261**  p=0.536** | r=-0.090*  p=0.847*  r=-0.347**  p=0.399** | r=-0.107*  p=0.839*  r=0.035**  p=0.964** | r=-0.643*  p=0.139*  r=-0.395**  p=0.322** | r=0.375*  p=0.252*  r=0.261**  p=0.498** | r=0.254*  p=0.451*  r=0.410**  p=0.273** | r=0.290*  p=0.386*  r=-0.224**  p=0.563** | r=0.183*  p=0.589*  r=0.447**  p=0.227** |

* - in non-survivors, ** - in survivors
