# Supplementary material for: Fraction of plasma exomeres and low-density lipoprotein cholesterol as a predictor of fatal outcome of COVID-19
Source: PLoS One. 2023 Feb 9;18(2):e0278083. doi: 10.1371/journal.pone.0278083 (PMC9910704; doi:10.1371/journal.pone.0278083)
Supplement: S5 Table — (DOCX) [file pone.0278083.s009.docx]

**S5 Table.** **The association between studied parameters of lipid parameters measured by DLS and standard technics for patients with COVID-19**

| **Predictors** | **At time to admission to the ICU** | | | **7 days after to admission to the ICU** | | |
| --- | --- | --- | --- | --- | --- | --- |
|  | **Odds Ratio** | **95% CI** | **p-value** | **Odds Ratio** | **95% CI** | **p-value** |
| **All patients with COVID-19** | | | | | | |
| **TC** | | | | | | |
| Sex | 1.001 | 0.241-4.164 | 0.9980 | 1.180 | 0.148-9.415 | 0.8757 |
| Age | 0.938 | 0.870-1.010 | 0.0904 | 0.932 | 0.846-9.415 | 0.1550 |
| Obesity | 0.144 | 0.023-0.898 | **0.0379** | 0.357 | 0.031-4.140 | 0.4102 |
| Hypertension | 0.525 | 0.112-2.465 | 0.4145 | 1.001 | 0.139-7.230 | 0.9993 |
| TC | 2.583 | 1.283- 5.206 | **0.0079** | 4.174 | 1.406-12.390 | **0.0101** |
| **HDL** | | | | | | |
| Sex | 1.367 | 0.375-4.988 | 0.636 | 0.917 | 0.158-5.314 | 0.9228 |
| Age | 0.944 | 0.881-1.012 | 0.102 | 0.908 | 0.826-0.999 | **0.0470** |
| Obesity | 0.130 | 0.023-0.731 | **0.021** | 0.154 | 0.015-1.638 | 0.1210 |
| Hypertension | 0.592 | 0.141-2.488 | 0.474 | 1.604 | 0.271-9.505 | 0.6025 |
| HDL | 5.642 | 0.599-53.114 | 0.130 | 49.939 | 1.014-2458.934 | **0.0492** |
| **LDL** | | | | | | |
| Sex | 1.074 | 0.255-4.518 | 0.9226 | 1.442 | 0.161-12.919 | 0.7436 |
| Age | 0.916 | 0.843-0.996 | **0.0392** | 0.923 | 0.833-1.021 | 0.1218 |
| Obesity | 0.169 | 0.027-1.072 | 0.0592 | 0.781 | 0.056-10.875 | 0.8540 |
| Hypertension | 0.540 | 0.112-2.616 | 0.4442 | 0.621 | 0.081-4.756 | 0.6464 |
| LDL | 3.385 | 1.351-8.482 | **0.0093** | 7.752 | 1.729-34.761 | **0.0075** |
| **TG** | | | | | | |
| Sex | 1.528 | 0.417- 5.605 | 0.5222 | 0.485 | 0.103-2.286 | 0.3602 |
| Age | 0.943 | 0.878-1.013 | 0.1110 | 0.923 | 0.847-1.007 | 0.0700 |
| Obesity | 0.137 | 0.025-0.756 | **0.0224** | 0.129 | 0.014-1.162 | 0.0678 |
| Hypertension | 0.737 | 0.185-2.938 | 0.6650 | 1.545 | 0.296-8.053 | 0.6055 |
| TG | 0.794 | 0.381-1.653 | 0.5369 | 1.678 | 0.638-4.410 | 0.2941 |
| **ExoM** | | | | | | |
| Sex | 0.667 | 0.137-3.257 | 0.6170 | 0.591 | 0.123-2.852 | 0.5128 |
| Age | 0.930 | 0.856-1.011 | 0.0905 | 0.921 | 0.846-1.002 | 0.0552 |
| Obesity | 0.158 | 0.023-1.088 | 0.0609 | 0.073 | 0.006-0.814 | **0.0334** |
| Hypertension | 0.422 | 0.079-2.243 | 0.3116 | 1.513 | 0.296-7.737 | 0.6187 |
| ExoM | 1.877e+30 | 5.259e+10-6.701e+49 | **0.0024** | 1.394 | 1.066e-05-1.823e+05 | 0.9560 |
| **LDL, rlpU** | | | | | | |
| Sex | 1.961 | 0.445-8.650 | 0.3739 | 1.576 | 0.155-1.601 | 0.7007 |
| Age | 0.945 | 0.875-1.020 | 0.1442 | 0.917 | 0.828-1.016 | 0.0967 |
| Obesity | 0.116 | 0.018-0.756 | **0.0244** | 0.253 | 0.014-4.719 | 0.3568 |
| Hypertension | 0.450 | 0.090-2.244 | 0.3298 | 0.490 | 0.048-4.977 | 0.5465 |
| LDL, rlpU | 1.941e+12 | 1.298e+04-2.901e+20 | **0.0032** | 4.373251e+23 | 6.376020e+06 2.999571e+40 | **0.0059** |
| **ExoM_LDL** | | | | | | |
| Sex | 1.287 | 0.244-6.780 | 0.7659 | 0.726 | 0.151-3.500 | 0.6900 |
| Age | 0.936 | 0.854-1.027 | 0.1607 | 0.930 | 0.856-1.001 | 0.0846 |
| Obesity | 0.096 | 0.011-0.868 | **0.0370** | 0.094 | 0.008-1.034 | 0.0533 |
| Hypertension | 0.633 | 0.101-3.941 | 0.6245 | 1.532 | 0.289-8.119 | 0.6159 |
| ExoM_LDL | 1.574e+21 | 1.515e+09-1.635e+33 | **0.0005** | 0.005 | 0.0001-6.724e+10 | 0.3106 |
| **ExoS** | | | | | | |
| Sex | 1.593 | 0.425-5.969 | 0.4898 | 0.661 | 0.126-3.462 | 0.6242 |
| Age | 0.941 | 0.878-1.009 | 0.0860 | 0.891 | 0.809-0.982 | **0.0197** |
| Obesity | 0.110 | 0.018-0.666 | **0.0163** | 0.056 | 0.004-0.852 | **0.0379** |
| Hypertension | 0.611 | 0.148-2.528 | 0.4968 | 2.002 | 0.349-1.148 | 0.4359 |
| ExoS | 7.438e-41 | 8.544e-84-6.475e+02 | 0.0670 | 9.886611e-48 | 6.493e-98-1.505e+03 | 0.0664 |
| **Patients with COVID-19 infected by alpha variant** | | | | | | |
| **TC** | | | | | | |
| Sex | 1.414 | 0.906-2.207 | 0.1430 | 1.630 | 0.934-2.842 | 0.1108 |
| Age | 0.983 | 0.961-1.005 | 0.1506 | 0.991 | 0.966-1.017 | 0.5053 |
| Obesity | 0.649 | 0.382- 1.101 | 0.1246 | 0.833 | 0.395-1.758 | 0.6407 |
| Hypertension | 1.405 | 0.848-2.329 | 0.2018 | 1.631 | 0.962-2.764 | 0.0942 |
| TC | 1.204 | 0.986-1.469 | 0.0837 | 1.191 | 1.032-1.374 | **0.0335** |
| **HDL** | | | | | | |
| Sex | 1.549 | 0.973- 2.464 | 0.0800 | 1.5372 | 0.783-3.016 | 0.2350 |
| Age | 0.980 | 0.958- 1.004 | 0.1170 | 0.9791 | 0.952-1.007 | 0.1670 |
| Obesity | 0.621 | 0.357-1.082 | 0.1080 | 0.7245 | 0.299-1.755 | 0.4890 |
| Hypertension | 1.475 | 0.867-2.510 | 0.1670 | 1.7489 | 0.932-3.286 | 0.1080 |
| HDL | 1.384 | 0.727-2.635 | 0.3340 | 1.6575 | 0.379-7.236 | 0.5140 |
| **LDL** | | | | | | |
| Sex | 1.372 | 0.877-2.149 | 0.1818 | 1.588 | 0.929-2.716 | 0.1192 |
| Age | 0.981 | 0.960-1.004 | 0.1291 | 0.995 | 0.970-1.021 | 0.7313 |
| Obesity | 0.625 | 0.369-1.057 | 0.0959 | 0.955 | 0.455-2.005 | 0.9061 |
| Hypertension | 1.392 | 0.840-2.308 | 0.2148 | 1.487 | 0.869-2.544 | 0.1757 |
| LDL | 1.232 | 0.964-1.575 | 0.1113 | 1.329 | 1.068-1.653 | **0.0265** |
| **TG** | | | | | | |
| Sex | 1.521 | 0.954-2.424 | 0.0932 | 1.546 | 0.805-2.968 | 0.2152 |
| Age | 0.980 | 0.957-1.004 | 0.1128 | 0.982 | 0.955-1.010 | 0.2317 |
| Obesity | 0.594 | 0.338-1.043 | 0.0847 | 0.651 | 0.276-1.538 | 0.3471 |
| Hypertension | 1.477 | 0.865- 2.523 | 0.1687 | 1.794 | 0.964-3.337 | 0.0898 |
| TG | 0.910 | 0.729-1.137 | 0.4171 | 1.145 | 0.876-1.496 | 0.3416 |
| **ExoM** | | | | | | |
| Sex | 1.329 | 0.892-1.981 | 0.1768 | 1.503 | 0.755-2.991 | 0.2690 |
| Age | 0.981 | 0.961- 1.000 | 0.0676 | 0.980 | 0.952-1.010 | 0.2130 |
| Obesity | 0.772 | 0.473-1.259 | 0.3123 | 0.704 | 0.288- 1.721 | 0.4560 |
| Hypertension | 1.239 | 0.782-1.961 | 0.3717 | 1.742 | 0.918- 3.307 | 0.1150 |
| ExoM | 5.515e+04 | 54.233-5.608e+07 | **0.0058** | 1.646 | 0.093-29.031 | 0.7400 |
| **LDL, rlpU** | | | | | | |
| Sex | 1.563 | 1.015-2.409 | 0.0564 | 0.974 | 0.968-2.561 | 0.0924 |
| Age | 0.981 | 0.960-1.003 | 0.1089 | 0.981 | 0.969-1.013 | 0.4469 |
| Obesity | 0.7420 | 0.347- 0.980 | **0.0552** | 0.8560 | 0.554-1.323 | 0.4939 |
| Hypertension | 1.413 | 0.861-2.320 | 0.1867 | 0.832 | 0.972-2.479 | 0.0906 |
| LDL, rlpU | 82.115 | 1.181-5707.771 | 0.055 | 1609.718 | 18.879-1.125e+05 | **0.0065** |
| **ExoM_LDL** | | | | | | |
| Sex | 1.474 | 0.977-2.225 | 0.0790 | 1.570 | 0.801- 3.077 | 0.2140 |
| Age | 0.982 | 0.961-1.003 | 0.1001 | 0.983 | 0.955-1.013 | 0.2850 |
| Obesity | 0.670 | 0.407-1.101 | 0.1294 | 0.741 | 0.307-1.788 | 0.5170 |
| Hypertension | 1.335 | 0.829-2.150 | 0.2482 | 1.748 | 0.937-3.261 | 0.1050 |
| ExoM_LDL | 577.776 | 4.473-74621.069 | **0.0185** | 3.355 | 0.191-58.957 | 0.4240 |
| **ExoS** | | | | | | |
| Sex | 1.538 | 0.697- 2.448 | 0.0843 | 1.472 | 0.785-2.759 | 0.2514 |
| Age | 0.983 | 0.960-1.007 | 0.1812 | 0.978 | 0.951-1.005 | 0.1297 |
| Obesity | 0.598 | 0.342-1.044 | 0.0855 | 0.653 | 0.282-1.512 | 0.3394 |
| Hypertension | 1.402 | 0.818-2.401 | 0.2332 | 1.732 | 0.949-3.164 | 0.0991 |
| ExoS | 2.652e-06 | 8.337e-18 8.440e+05 | 0.3534 | 1.662e-06 | 1.226e-15 2.254e+03 | 0.2385 |
| **Patients with COVID-19 infected by delta variant** | | | | | | |
| **TC** | | | | | | |
| Sex | 1.005 | 0.728-1.387 | 0.9761 | 0.881 | 0.538-1.441 | 0.6208 |
| Age | 0.997 | 0.979-1.006 | 0.2920 | 0.988 | 0.972-1.004 | 0.1522 |
| Obesity | 0.752 | 0.537-1.052 | 0.1063 | 0.981 | 0.635-1.516 | 0.9332 |
| Hypertension | 0.715 | 0.516-0.991 | 0.0522 | 0.954 | 0.597-1.527 | 0.8483 |
| TC | 1.128 | 0.984-1.292 | 0.0931 | 1.232 | 1.100-1.380 | **0.0026** |
| **HDL** | | | | | | |
| Sex | 1.053 | 0.773-1.434 | 0.7446 | 0.855 | 0.507-1.440 | 0.5646 |
| Age | 0.992 | 0.978- 1.000 | 0.2401 | 0.984 | 0.968-1.000 | 0.0819 |
| Obesity | 0.737 | 0.527-1.032 | 0.0849 | 0.835 | 0.535-1.303 | 0.4394 |
| Hypertension | 0.694 | 0.498-0.967 | **0.0388** | 1.087 | 0.665-1.781 | 0.7400 |
| HDL | 1.591 | 0.886- 2.857 | 0.1299 | 2.401 | 1.389-4.152 | **0.0068** |
| **LDL** | | | | | | |
| Sex | 1.043 | 0.765-1.423 | 0.7901 | 0.983 | 0.969-0.998 | 0.9437 |
| Age | 0.990 | 0.976-1.005 | 0.1977 | 0.983 | 0.618-1.564 | **0.0372** |
| Obesity | 0.760 | 0.541-1.069 | 0.1252 | 1.115 | 0.732-1.698 | 0.6201 |
| Hypertension | 0.729 | 0.525-1.013 | 0.0691 | 0.828 | 0.528-1.297 | 0.4228 |
| LDL | 1.141 | 0.973-1.337 | 0.1137 | 1.456 | 1.222-1.734 | **0.0008** |
| **TG** | | | | | | |
| Sex | 1.138 | 0.830-1.561 | 0.4271 | 0.771 | 0.401-1.485 | 0.4490 |
| Age | 0.994 | 0.978-1.010 | 0.4527 | 0.985 | 0.964-1.007 | 0.2050 |
| Obesity | 0.715 | 0.504-1.012 | 0.0675 | 0.786 | 0.450-1.374 | 0.4120 |
| Hypertension | 0.720 | 0.511-1.014 | 0.0694 | 1.112 | 0.598-2.068 | 0.7420 |
| TG | 0.991 | 0.836-1.174 | 0.9153 | 1.057 | 0.926- 1.207 | 0.4260 |
| **ExoM** | | | | | | |
| Sex | 1.079 | 0.803-1.450 | 0.6170 | 1.425 | 0.506-1.558 | 0.6840 |
| Age | 0.997 | 0.983-1.011 | 0.6622 | 0.987 | 0.967-1.007 | 0.2260 |
| Obesity | 0.753 | 0.538-1.053 | 0.1070 | 0.642 | 0.367-1.123 | 0.1400 |
| Hypertension | 0.716 | 0.517-0.990 | **0.0517** | 1.080 | 0.614-1.901 | 0.7930 |
| ExoM | 1.046 | 0.990-1.106 | 0.1189 | 1.051 | 0.969-1.138 | 0.2470 |
| **LDL, rlpU** | | | | | | |
| Sex | 1.099 | 0.824-1.464 | 0.5260 | 0.974 | 0.645-1.471 | 0.9013 |
| Age | 0.994 | 0.981-1.007 | 0.3591 | 0.981 | 0.967-0.996 | **0.0231** |
| Obesity | 0.742 | 0.535-1.029 | 0.0827 | 0.856 | 0.5539- 1.323 | 0.4939 |
| Hypertension | 0.718 | 0.521-0.989 | 0.0507 | 0.832 | 0.546-1.267 | 0.4043 |
| LDL, rlpU | 28.926 | 0.886-944.382 | 0.0673 | 1609.718 | 45.302- 5.720e+04 | **0.0009** |
| **ExoM_LDL** | | | | | | |
| Sex | 1.009 | 0.774-1.315 | 0.9467 | 0.891 | 0.547-1.450 | 0.6470 |
| Age | 1.001 | 0.989-1.014 | 0.8462 | 0.988 | 0.971-1.005 | 0.1870 |
| Obesity | 0.808 | 0.598-1.091 | 0.1727 | 0.723 | 0.547-1.450 | 0.2270 |
| Hypertension | 0.753 | 0.564-1.006 | 0.0638 | 1.043 | 0.642-1.693 | 0.8680 |
| ExoM_LDL | 1.075 | 1.032-1.120 | **0.0015** | 1.089 | 1.023-1.160 | **0.0170** |
| **ExoS** | | | | | | |
| Sex | 1.083 | 0.794-1.477 | 0.6190 | 0.949 | 0.524-1.718 | 0.8640 |
| Age | 0.993 | 0.981-1.007 | 0.3590 | 0.982 | 0.962-1.004 | 0.1330 |
| Obesity | 0.742 | 0.522-1.054 | 0.1050 | 0.662 | 0.362-1.212 | 0.2000 |
| Hypertension | 0.752 | 0.531-1.065 | 0.1180 | 1.071 | 0.588-1.952 | 0.8250 |
| ExoS | 2.898e-05 | 5.247e-15-1.601e+05 | 0.3680 | 5.927e-06 | 5.367e-24-6.547e+12 | 0.5780 |
