# Supplementary material for: Fraction of plasma exomeres and low-density lipoprotein cholesterol as a predictor of fatal outcome of COVID-19
Source: PLoS One. 2023 Feb 9;18(2):e0278083. doi: 10.1371/journal.pone.0278083 (PMC9910704; doi:10.1371/journal.pone.0278083)
Supplement: S6 Table — (DOCX) [file pone.0278083.s010.docx]

**S6 Table.** **mRNA levels of the studied genes in PBMCs of patients with severe COVID-19 with Delta variant and in the control group.**

| Genes | Relative expression level of the studied genes | | | | |
| --- | --- | --- | --- | --- | --- |
|  | Admission to the ICU | | 7 days after admission to the ICU | |  |
|  | Non-survivors (N=13) | Survivors (N=15) | Non-survivors (N=13) | Survivors (N=15) | Control group (N= 20) |
| *PPARG* | 2.55(0.07 -15.82)  **p=0.00024*** | 1.62(0.15-20.63)  **p=0.00048*** | 0.07 (0.01-0.55)  **p=0.016****** | 0.171 (0.05-1.66)  **p=0.016***** | 0.277(0.02 -0.97) |
| *LDLR* | 1.30(0.035-3.37) | 1. 00(0.027-4.55)  **p=0.0047*** | 2.99 (0.83-8.52)  **p<0.0001*** | 1.92 (0.29-6.654)  **p<0.0001*** | 0.20(0.01-0.44) |
| *LRP6* | 0.91(0.19-201.04) | 1.51(0.26-13.88) | 0.613 (0.01-18.5) | 0.05 (0.01-1.25)  **p=0.0089***  **p=0.02***** | 0.70(0.09-7.91) |
| *ANXA2* | 0.28(0.04-2.36) | 0.48 (0.01-5.58)  **p=0.011*** | 0.05 (0.03-0.11)  p=0.0011*  **p=0.00098****** | 0.021 (0.03-0.056)  **p=0.02***  **p=0.0029***** | 0.18(0.04-2.57) |
| *STAB1* | 0.42(0.065-2.39) | 0.55(0.13- 21.32) | 21.032 (1.12-45.852)  **p<0.05***  **p=0.017****  **p =0.0078****** | 13.630 (5.92-25.245)  **p<0.05*** | 0.78(0.05-7.27) |
| *CD36* | 2.359(0.15-19.15)  **p=0.0027*** | 5.58(0.32-16.49)  **p<0.0001*** | 2.565 (0.27-13.04)  **p<0.05*** | 1.84 (0.07-25.247)  **p<0.05*** | 0.34(0.04-3.68) |

* - compared with control, ** - compared with survivors with COVID-19 (7 days after admission to ICU), *** - compared with survivors with COVID-19 (admission to ICU), **** - compared with Non-survivor with COVID-19 (admission to ICU)
