# Supplementary material for: Fraction of plasma exomeres and low-density lipoprotein cholesterol as a predictor of fatal outcome of COVID-19
Source: PLoS One. 2023 Feb 9;18(2):e0278083. doi: 10.1371/journal.pone.0278083 (PMC9910704; doi:10.1371/journal.pone.0278083)
Supplement: Attachment — Submitted filename: Response to Reviewers.docx [file pone.0278083.s011.docx]

Dear reviewers, we greatly appreciate all comments.

**Reviewer #1:**

1. The introduction contains parts of the method section. Only relevant information should be presented in the introduction.

- Was shortened and corrected.

2. Have patients' medications been considered? lipid-lowering agents, especially PCSK9 inhibitors.

- As the inclusion criteria include the absence of chronic comorbidities such as cancer, cerebrovascular diseases, heart failure, or renal failure all patients included in the study did not receive the lipid-lowering therapy. This information was included in the text.

3. Why was the evaluation of the patients made after 7 days?

This day was chosen as the first point suitable for assessing the effectiveness of pathogenic therapy. The average time a patient spends in the intensive care unit of Pavlov First Saint-Petersburg State Medical University is 7 days.

4. How was the number of individuals enrolled in the study determined? Was the sample size calculated?

All patients admitted to the intensive care unit and met the inclusion criteria were included in the study. Blood samples were collected in two waves, during an infection with alpha and delta variants of SARS-CoV-2 virus. This information was added in the text.

**Reviewer #2:** In this study, the authors assessed the relationship between cholesterol metabolism and clinical outcome of patients with severe COVID-19. They show that a downward trend of cholesterol is associated with poorer prognosis.

The title of the paper suggests that the study is about cholesterol metabolism and outcome, but we are also given lots of information about cytokine levels and the correlation between lipid profiles measured with DLS and standard techniques. All this moves the attention a bit away from the main message: downregulation of cholesterol and prognosis. The authors need a lot of space to make their point and mix a lot of data. This makes the paper difficult to read. Why not describing the relationship between cholesterol fractions and prognosis (with more patients, of course) as a clinically relevant observation. All the other things are more mechanistic in nature and will not immediately interest the practicing physician.

Thank you very much. The title of the article has been changed and the main body of the article restructured in order to strengthen the link of blood cholesterol fractions and COVID-19 prognosis.

The paper needs considerable language polishing.

Introduction
This part of the paper is unduly long and not so easy to read. It is not focused well on the problem which needs to be studied. It also lacks a clear hypothesis.

-The Introduction section was shortened and the following supposition was added “We suppose that exomers may play a role in COVID-19 pathogenesis as an essential role of exomeres in cholesterol transport was shown. To further analyze the role of cholesterol metabolism in poor prognosis of COVID-19 we evaluate retrospectively the parameters of cholesterol metabolism (plasma lipids concentrations and exomere fraction, the expression levels of LDLR activity pathway genes selected through our previous transcriptome analysis) as well as cytokine profile as possible predictors of fatal outcome in patients with severe COVID-19 infected by alpha and delta variants of SARS-CoV-2 virus”.

Methods
The authors should clarify the approval by the Ethics Board in more detail. The present study is a retrospective analysis, but all participants apparently signed a written consent prior to the study. How is that possible when patients died? This is particularly relevant with respect to the replication cohort.

-The written concern was signed in the time of admission in the intensive care unit.

Were all patients admitted in July and August included in the study or was there some selection?

-All patients admitted to the intensive care unit and met the inclusion criteria were included in the study. This information was added in the text.

The replication study included patients had had been admitted several months before the main study. This is odd because a replication study usually follows the main study and needs to have a prospective design. Here, the replication study is a retrospective analysis as well with all its inherent shortcomings and can, therefore, not truly serve as a replication.

-We made a correction and aggregated the study in one with a comparison between patients with COVID-19 infected by еру Alpha and Delta variants of SARS-CoV-2 virus. It allows us to conduct a comparison between analyzed marker for poor COVID-19 prognosis among patients affected with different variants of SARS-CoV-2 virus. Particularly, an information about an association of the Delta variant with a more pronounce decrease in LDL levels in non-surviving patients during the time spent in the ICU is discussed.
